# Supplementary material for: Hybrid Models and Biological Model Reduction with PyDSTool
Source: PLoS Comput Biol. 2012 Aug 9;8(8):e1002628. doi: 10.1371/journal.pcbi.1002628 (PMC3415397; doi:10.1371/journal.pcbi.1002628)
Supplement: Text S4 — Complete source code for the PyDSTool package (version 0.88.120504). Includes API documentation and help files linking to web pages. This file is identical to the current public release on Sourceforge.net. (ZIP) [file pcbi.1002628.s004.zip › PyDSTool/html/PyDSTool.PyCont.TestFunc.TestFunc-class.html]

xml version="1.0" encoding="ascii"?


PyDSTool.PyCont.TestFunc.TestFunc


| Home | Trees | Indices | Help | PyDSTool |
| --- | --- | --- | --- | --- |

Package PyDSTool ::
Package PyCont ::
Module TestFunc ::
Class TestFunc

[hide private]

[frames] | no frames]

# Class TestFunc

source code

```
object --+    
         |    
  Function --+
             |
            TestFunc
```

Known Subclasses:
:   - BT\_Fold
    - , BT\_Hopf
    - , BT\_Hopf\_One
    - , B\_Check
    - , BiAltMethod
    - , BorderMethod
    - , Branch\_Det
    - , CP\_Fold
    - , DH\_Hopf
    - , Fold\_Det
    - , Fold\_Tan
    - , GH\_Hopf
    - , GH\_Hopf\_One
    - , Hopf\_Eig
    - , LPC\_Det
    - , PD\_Det
    - , ParTestFunc
    - , UserDefinedTestFunc

---

You need to define the function yourself within an inherited
class.


|  |  |
| --- | --- |
| Instance Methods | [hide private] |

|  |  |
| --- | --- |
| \_\_init\_\_(self, (n, m), F, C, save=False, numpoints=None)  x.\_\_init\_\_(...) initializes x; see x.\_\_class\_\_.\_\_doc\_\_ for signature | source code |

|  |  |
| --- | --- |
| findzero(self, P1, P2, ind)  Uses secant method to find zero of test function. | source code |

|  |  |
| --- | --- |
| diff(self, x0, ind=None)  This is recreated from Function class above for TestFunc class. | source code |

**Inherited from `Function`**:
`__call__`,
`__getitem__`,
`__setitem__`,
`numhess`

**Inherited from `object`**:
`__delattr__`,
`__getattribute__`,
`__hash__`,
`__new__`,
`__reduce__`,
`__reduce_ex__`,
`__repr__`,
`__setattr__`,
`__str__`


|  |  |
| --- | --- |
| Properties | [hide private] |

**Inherited from `object`**:
`__class__`


|  |  |
| --- | --- |
| Method Details | [hide private] |

|  |  |
| --- | --- |
| \_\_init\_\_(self, (n, m), F, C, save=False, numpoints=None)  *(Constructor)* | source code |

x.\_\_init\_\_(...) initializes x; see x.\_\_class\_\_.\_\_doc\_\_ for
signature

Overrides: object.\_\_init\_\_: *(inherited documentation)*

|  |  |
| --- | --- |
| findzero(self, P1, P2, ind) | source code |

Uses secant method to find zero of test function.

Overrides: Function.findzero

|  |  |
| --- | --- |
| diff(self, x0, ind=None) | source code |

This is recreated from Function class above for TestFunc class. Why,
you ask? Because for test functions, the jacobian of the parent function
F needs to be computed before every test function call. Unforunately,
this slows things down, but that is unavoidable.

Overrides: Function.diff

  


| Home | Trees | Indices | Help | PyDSTool |
| --- | --- | --- | --- | --- |

|  |  |
| --- | --- |
| Generated by Epydoc 3.0.1 on Fri May 4 15:24:07 2012 | http://epydoc.sourceforge.net |
